# Ilizarov-assisted periosteal distraction for refractory arteriovenous malformation ulcers: first clinical report in Asia

**DOI:** 10.1186/s41065-025-00479-4

**Published:** 2025-06-18

**Authors:** Xiangyi Wu, Ren Cai, Mao Ye, Xitao Yang, Dachuan Sun, Yifeng Han, Xindong Fan, Jiaxue Zhu

**Affiliations:** 1 Department of Interventional Therapy, Multidisciplinary Team of Vascular Anomalies, Shanghai Ninth People’s Hospital, Shanghai Jiao Tong University School of Medicine, Shanghai, PR China; 2 Department of Plastic and Reconstructive Surgery, Shanghai Ninth People’s Hospital, Shanghai Jiao Tong University School of Medicine, Shanghai, PR China; 3 Department of Orthopedics, Fengcheng Hospital Affiliated to Shanghai Ninth People’s Hospital, Shanghai Jiao Tong University, Shanghai, PR China

**Keywords:** Extracranial arteriovenous malformation, Ilizarov technique, Periosteal distraction, Ulcer healing, Chemotherapy

## Abstract

Refractory ulcers caused by high-flow arteriovenous malformations (AVMs) pose significant therapeutic challenges due to persistent tissue ischemia and shear stress-induced graft failure. Traditional embolization or flap reconstruction strategies often yield suboptimal outcomes, particularly in weight-bearing regions. We present a 28-year-old female with a non-healing dorsal foot AVM ulcer despite multiple embolizations and radical toe amputations. Genetic testing revealed a KRAS mutation, confirming a somatic etiology of AVM. After a debridement and local flap repair, we employed Ilizarov-assisted periosteal distraction to improve local perfusion. 75% of the wound epithelialized and the infection resolved within 4 weeks postoperatively, with granulation tissue covering the remainder. This case highlights the mechanobiological advantages of Ilizarov-based neovascular stimulation in AVM-related ischemic ulcers.

## Introduction

 Extremity AVMs account for 15–20% of congenital vascular anomalies [1], with nearly one-third progressing to chronic ulceration due to persistent arteriovenous shunting and resultant tissue hypoxia [2]. In addition to local ischemia, turbulent flow and shear stress within affected vascular beds contribute to poor graft take and wound recurrence, especially in the foot [3]. Although superselective embolization and tissue flap coverage are mainstays, their efficacy is often limited in weight-bearing zones or when nidus involvement is extensive [4].

Mechanobiological interventions, such as Ilizarov-based periosteal distraction, offer an innovative strategy to overcome microcirculatory deficits [5]. Inspired by distraction osteogenesis [6], this technique uses gradual mechanical tension to stimulate angiogenesis via VEGF upregulation and periosteal stem cell activation. We report the first case of Ilizarov-assisted wound healing in an AVM-related ulcer with a known KRAS6 mutation.

### Report

A 28-year-old female presented to our hospital with a non-healing 5 × 4 cm ulcer on the dorsal aspect of the right foot. The lesion was overlying a diffuse, infiltrative Shobinger stage III AVM confirmed by angiography. Genetic analysis demonstrated a somatic KRAS mutation, supporting the diagnosis of congenital AVM.

**Fig. 1:**
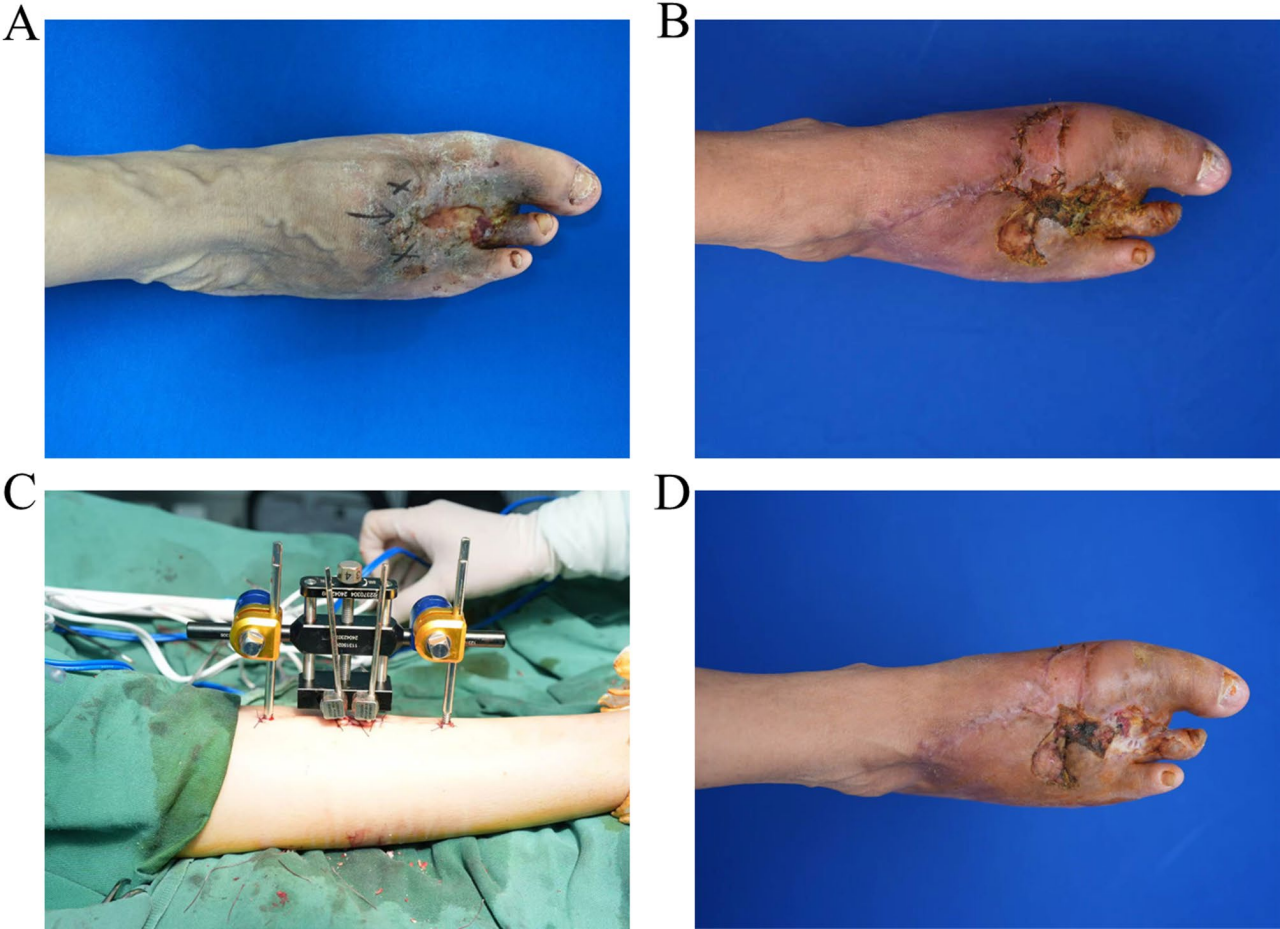
Clinical presentation and intraoperative findings. (**A**) Preoperative photograph showing a chronic ulcer on the dorsum of the right foot measuring approximately 5 × 4 cm. The surrounding skin is pale and poorly perfused. (**B**) Wound after initial debridement and flap grafting. (**C**) Placement of the Ilizarov ring fixator and insertion of percutaneous Kirschner wires in the proximal and distal tibia for periosteal traction. (**D**) Four-week postoperative follow-up showing significant epithelialization and granulation tissue formation throughout the wound bed

Before admission, the patient underwent three sessions of Onyx embolization over the past decade, followed by repeated coil extrusion and skin breakdown episodes. In 2021, due to persistent ischemia and ulceration with exposed embolic material, she underwent radical amputation of the second and fourth toes. Despite temporary wound stabilization, new ulcerations developed over the dorsum and medial midfoot within 6 months postoperatively, complicated by methicillin-sensitive Staphylococcus aureus (MSSA) infection (Fig. 1A). Reluctant to undergo a full foot amputation, the patient sought further evaluation at our institution. On arrival, clinical examination revealed active drainage, poor perfusion, and exposed tendon without bone involvement. Following thorough debridement and local transposition flap coverage (Fig. 1B), we planned staged revascularization using the Ilizarov technique.

Four 1.8 mm tensioned Kirschner wires were inserted into the proximal and distal tibia, secured via a circular fixator (Fig. 1C). Starting on postoperative day 5, periosteal distraction was initiated at 0.25 mm twice daily for 20 days. At 4 weeks, 75% of the area was re-epithelialized and granulation tissue covered the entire wound base. The skin surrounding the wound appeared erythematous with adequate perfusion (Fig. 1D). In addition, infection was controlled without the need for systemic antibiotic. At the 12-month follow-up, the patient resumed independent ambulation with no ulcer recurrence, reported minimal pain, and returned to normal daily activities.

## Discussion

Chronic ulcers associated with AVMs are notoriously difficult to treat due to the complex interplay of ischemia, high-flow shunting, and mechanical stress [7]. KRAS mutations, now recognized as key somatic drivers of AVM pathogenesis, further implicate aberrant endothelial proliferation and vessel remodeling in disease progression [8, 9]. These molecular aberrations contribute to unstable vasculature, limited healing, and recurrent ulceration, as demonstrated in our patient [10].

Ilizarov periosteal distraction represents a novel mechanotherapeutic approach, particularly suited to ischemia-related wounds [11, 12]. Unlike standard revascularization or graft-based solutions, it leverages the body’s own regenerative pathways. Mechanical tension promotes upregulation of angiogenic factors (e.g., VEGF, PDGF), recruits mesenchymal stem cells, and enhances capillary sprouting from the inner periosteum [13, 14]. Moreover, the external fixator offloads pressure from the ulcer bed, reducing mechanical shear, a critical factor in our patient’s failure of previous grafts.

Compared with microvascular flap reconstruction, which provides immediate tissue coverage, the Ilizarov method is less invasive and better suited for compromised vascular beds. Flap-based interventions frequently fail in AVM-afflicted limbs due to inadequate perfusion and turbulent flow within the nidus [15]. Similarly, negative pressure wound therapy (NPWT) may enhance granulation and manage exudate, yet remains limited in the absence of underlying vascular restoration [16]. In contrast, the Ilizarov technique directly addresses the ischemic pathophysiology through localized angiogenesis and mechanical offloading, making it particularly advantageous for weight-bearing or previously embolized regions.

This case reinforces the feasibility and effectiveness of Ilizarov-assisted vascular stimulation in the management of AVM ulcers. Periosteal distraction may serve as a bridging therapy to avoid major amputation and promote durable closure in otherwise refractory wounds.

## Data Availability

No datasets were generated or analysed during the current study.
